# Supplementary material for: Proteome-wide C-degron activity profiling connects conditional regulation of the CTLH E3 ligase complex to ribosome biogenesis
Source: bioRxiv. 2026 Jan 14:2026.01.14.698769. Preprint. [Version 1] doi: 10.64898/2026.01.14.698769 (PMC12871134; doi:10.64898/2026.01.14.698769)
Supplement: Supplement 1 [file NIHPP2026.01.14.698769v1-supplement-1.pdf]

bioRxiv preprint doi: https://doi.org/10.64898/2026.01.14.698769; this version posted January 14, 2026. The copyright holder for this preprint (which was not certified by peer review) is the author/funder, who has granted bioRxiv a license to display the preprint in perpetuity. It is made available under aCC-BY 4.0 International license.

SUPPLEMENTARY FIGURE LEGENDS

Supplementary Figure 1 | Comparative stability profiling identifies full-length human proteins bearing C-terminal degrons.

(A) Venn diagrams representing the overlap between the constituent members of the two ORFeome libraries.

(B) Schematic depicting the rationale underpinning the comparative screening approach. Should an E3 ligase target a substrate for ubiquitination and degradation via a degron located at its extreme C-terminus (left), we reasoned that "capping" of the C-terminus via the addition of an invariant 9 amino acids in the ORFeome V8.1 expression vector (right) would prevent E3 ligase recognition and result in stabilization of the substrate.

(C) Assessing the concordance between replicate screens. Each dot depicts the performance (ΔPSI, the difference in stability when assayed as part of the ORFeome V8.1 library compared to the Ultimate ORFeome library) of each protein detected in both libraries across duplicate experiments. Red dots indicate the 52 proteins exhibiting concordant stabilization >0.75 PSI units as part of the ORFeome V8.1 library.

(D) Assessing the screen performance of individually barcoded replicates for the selected substrates. Each ORF in the libraries is represented by an average of five independent barcodes, thus providing internal replicates; screen profiles (depicting the distribution of sequencing reads across the six stability bins) for each individually barcoded replicate are shown, when assayed as part of the Ultimate ORFeome (gray traces) compared to the ORFeome V8.1 (red traces).

bioRxiv preprint doi: https://doi.org/10.64898/2026.01.14.698769; this version posted January 14, 2026. The copyright holder for this preprint (which was not certified by peer review) is the author/funder, who has granted bioRxiv a license to display the preprint in perpetuity. It is made available under aCC-BY 4.0 International license.

Supplementary Figure 2 | Candidate proteins harboring C-terminal degrons.

Screen profiles are shown for all 52 proteins exhibiting concordant stabilization when assayed as part of the ORFeome V8.1 library as compared to the Ultimate ORFeome library across duplicate screens.

Supplementary Figure 3 | Defining the machinery required for the degradation of ZMYND19.

(A) Saturation mutagenesis of the ZMYND19 C-terminus yields highly concordance stability scores. The scatterplot reflects the protein stability index metric (1 = maximally unstable, 3 = maximally stable) for each ZMYND19 mutant across two replicate experiments.

(B) Ubiquitin-dependent proteasomal degradation of ZMYND19. HEK-293T cells expressing GPS-ZMYND19 were treated with the indicated inhibitors and assessed by flow cytometry 8 hours later. Treatment with the proteasome inhibitor Bortezomib and the E1 inhibitor TAK-243 resulted in ZMYND19 stabilization, whereas the pan-Cullin inhibitor MLN4924 and the lysosomal inhibitor Bafilomycin A1 did not.

(C) Schematic representation of a genome-wide CRISPR screen designed to identify the machinery required for ZMYND19 degradation.

(D-E) Validation of knockout clones lacking CTLH subunits generated by CRISPR/Cas9-mediated gene disruption. Validation was performed by immunoblot **(D)** with the exception of the GID4 KO clone, which, owing to the lack of effective commercial antibodies, was assessed by Illumina sequencing **(E)**.

(F) Assessing the stability of ZMYND19 in the KO clones lacking CTLH subunits. GPS-ZMYND19 was expressed in clones of the indicated genotypes and its stability assayed by flow cytometry.

bioRxiv preprint doi: https://doi.org/10.64898/2026.01.14.698769; this version posted January 14, 2026. The copyright holder for this preprint (which was not certified by peer review) is the author/funder, who has granted bioRxiv a license to display the preprint in perpetuity. It is made available under aCC-BY 4.0 International license.

(G) An HA-tagged ZMYND19 construct is more abundant in MAEA KO and Muskelin KO clones, as assessed by immunoblot.

Supplementary Figure 4 | Conditional regulation of ZYMND19 degradation.

(A) Reproducible effects of TNF-α stimulation and mTOR inhibition on ZMYND19 stability. HEK-293T cells expressing ZMYND19 in the context of the GPS expression vector were subjected to the indicated treatments in triplicate, and the effect on the stability of ZMYND19 assayed by flow cytometry.

(B) TNF-α stimulation does not broadly affect proteasomal degradation. HEK-293T cells were transduced with GPS vectors encoding ZMYND19 and CHGA, treated with TNF-α overnight and then analyzed by flow cytometry.

(C) Amino acid withdrawal or mTOR inhibition reproducibly overrides the effects of TNF-α and promotes ZMYND19 degradation. A replicate experiment of the one performed in Fig. 4E is shown.

(D) Assessing the effects of TNF-α stimulation and mTOR inhibition on CTLH complex assembly. CTLH was immunoprecipitated from HEK-293T cells treated with either TNF-α or Torin-1 using an antibody against RANBP9, and co-immunoprecipitating CTLH subunits assessed by immunoblot.

Supplementary Figure 5 | Identifying additional substrates of the CTLH^{Muskelin} C-degron pathway.

(A) Performance of individually barcoded replicates for the CTLH substrates identified by the stability profiling screen.

(B) A small reduction in stability is observed following overnight Torin-1 treatment when GFP alone is expressed in the context of the GPS vector.

bioRxiv preprint doi: https://doi.org/10.64898/2026.01.14.698769; this version posted January 14, 2026. The copyright holder for this preprint (which was not certified by peer review) is the author/funder, who has granted bioRxiv a license to display the preprint in perpetuity. It is made available under aCC-BY 4.0 International license.

(C) Purifying cells lacking CTLH subunits for proteomic analysis. HEK-293T cells expressing GPS-ZMYND19 were transduced in duplicate with Cas9 and sgRNAs targeting the indicated genes; cells lacking a functional CTLH complex stabilize ZMYND19 and can therefore be isolated by sorting for GFP^+ cells. Two intron-targeting sgRNAs were used in parallel as negative controls, which were also sorted in an equivalent manner (targeting $DsRed^+$/GFP^- cells).

(D) Overlay of the crystal structure of Sqt1 (pink) and the AlphaFold 3-predicted structure of AAMP (gold).

(E) Schematic representation of the ISG20 family of proteins.

(F) The interaction between Muskelin and AAMP is preserved following treatment with Torin-1 or TNF-α.

(G) The effect of Torin-1 treatment and Muskelin knockout on the abundance of endogenous AAMP, as assessed by immunoblot.

(H-J) The C-terminus of AAMP is recognized by the β-propellor formed by the kelch repeats of Muskelin. **(H)** Superimposition of AlphaFold 3 structure predictions suggests that Muskelin engages the C-termini of ZMYND19 and AAMP in an analogous manner. **(I)** Expression levels of the indicated FLAG-tagged Muskelin mutants as assessed by immunoblot. **(J)** Muskelin mutants affecting the predicted binding interface abolish the association between AAMP and Muskelin. The indicated FLAG-tagged Muskelin mutants were expressed in cells expressing GPS-AAMP, and binding assessed by immunoprecipitation with an anti-GFP nanobody followed by FLAG immunoblot.

bioRxiv preprint doi: https://doi.org/10.64898/2026.01.14.698769; this version posted January 14, 2026. The copyright holder for this preprint (which was not certified by peer review) is the author/funder, who has granted bioRxiv a license to display the preprint in perpetuity. It is made available under aCC-BY 4.0 International license.

Supplementary Figure 6 | Collateral degradation of the AAMP interacting partner AEN by the CTLH^{Muskelin} C-degron pathway.

(A) Validation of efficient shRNA-mediated depletion of AAMP. HEK-293T cells were transduced with lentiviral shRNA expression vectors and the extent of AAMP depletion four days post-transduction was measured by immunoblot. The two most efficient shRNAs, sh1-AAMP and sh3-AAMP, were used for subsequent experiments.

(B-D) AEN is subject to collateral degradation by CTLH^{Muskelin} via AAMP. **(B)** Schematic representation of the experimental setup to determine the impact of manipulating the C-terminus of AAMP on the CTLH-mediated degradation of AEN. Owing to the essentiality of AAMP (see Fig. 7), we performed the genetic complementation assay by first introducing shRNA-resistant AAMP constructs into cells expressing GPS-AEN, followed by shRNA-mediated knockdown of endogenous AAMP. **(C)** The association between AEN and Muskelin requires the C-terminus of AAMP. Immunoprecipitation of GFP-tagged AEN pulled-down Muskelin when wild-type AAMP was present, but to a lesser extent in the presence of mutant versions of AAMP bearing either a truncated (“del5”) or capped (“add5”) C-terminus. **(D)** The degradation of AEN stimulated by mTOR inhibition requires the C-terminus of AAMP. The indicated AAMP constructs were introduced into cells expressing GPS-AEN at single copy, as assessed by flow cytometry measurement of BFP^{+} cells (left panel). Following blasticidin selection (center panel), the cells were transduced with shRNA expression vectors to deplete endogenous AAMP, and, following overnight treatment with Torin-1, the stability of AEN was then assayed by flow cytometry (right panel).

(E-F) AAMP interacts with ISG20L2. **(E)** Co-immunoprecipitation of ISGL20L2 with AAMP, as detected by immunoblot. **(F)** AlphaFold 3 model of the ISG20L2-AAMP interaction, which suggests a basic α-helix located at the N-terminus ISG20L2 may be accommodated by an acidic pocket formed by the WD40 repeats of AAMP.

bioRxiv preprint doi: https://doi.org/10.64898/2026.01.14.698769; this version posted January 14, 2026. The copyright holder for this preprint (which was not certified by peer review) is the author/funder, who has granted bioRxiv a license to display the preprint in perpetuity. It is made available under aCC-BY 4.0 International license.

(G-H) ISG20L2 is not a substrate of collateral degradation by the CTLH^{Muskelin} C-degron pathway. The stability of ISG20L2 is not affected by Torin-1 treatment when expressed in the context of the GPS expression system **(G)**, and the abundance of endogenous ISG20L2 is not affected upon ablation of Muskelin **(H)**.

Supplementary Figure 7 | AAMP binds uL16 and ISG20L2 to regulate ribosome biogenesis.

(A) Sequence alignment of *S. cerevisiae* Sqt1 and *H. sapiens* AAMP. Blue shading reflects the degree of amino similarity. The acidic residues mutated in subsequent experiments are highlighted in red boxes.

(B-D) Recognition of uL16 by AAMP is necessary for cell viability. **(B)** Design of the AAMP "4K" charge-swap mutant. Analysis of the Sqt1-uL16 interface (left) highlights four acidic residues contacting uL16; the corresponding acidic residues are also predicted to contact uL16 in the AlphaFold 3 model of the AAMP-uL16 interaction (right). **(C)** The 4K charge-swap mutant of AAMP failed to associate with uL16, as assessed by co-immunoprecipitation experiments, and, unlike wild-type AAMP, could not restore cell viability upon knockdown of endogenous AAMP **(D)**.

(E) Reduced ISG20L2 abundance upon AAMP knockdown. As was the case for uL16 (Fig. 7J), AAMP depletion reduced ISG20L2 protein levels; this effect could be reversed upon exogenous expression of an shRNA-resistant wild-type AAMP, but not by the 4K charge-swap AAMP mutant.

bioRxiv preprint doi: https://doi.org/10.64898/2026.01.14.698769; this version posted January 14, 2026. The copyright holder for this preprint (which was not certified by peer review) is the author/funder, who has granted bioRxiv a license to display the preprint in perpetuity. It is made available under aCC-BY 4.0 International license.

Supplementary Figure 8 | NAP1L2 is a candidate substrate of the CTLH$^{\mathbf{Muskelin}}$ C-degron pathway.

(A) Scatterplot representing TMT proteomics data from. Substrates discussed in this study are shown in bold font.

(B) AlphaFold 3 prediction of the interaction between the C-terminus of NAP1L2 and the β-propellor formed by the kelch repeats of Muskelin.

(C) Superimposition of AlphaFold 3 structural predictions, showing that the C-terminus of NAP1L2 is predicted to contact the same residues in the β-propellor formed by the kelch repeats of Muskelin as ZMYND19 and AAMP.

bioRxiv preprint doi: https://doi.org/10.64898/2026.01.14.698769; this version posted January 14, 2026. The copyright holder for this preprint (which was not certified by peer review) is the author/funder, who has granted bioRxiv a license to display the preprint in perpetuity. It is made available under aCC-BY 4.0 International license.

SUPPLEMENTARY TABLE LEGENDS

Supplementary Table 1 | Comparative stability profiling screens to identify full-length proteins bearing C-terminal stability determinants. ORF-level data showing the corrected read counts across the six stability bins for each ORF detected common to both libraries across replicate experiments.

Supplementary Table 2 | Saturation mutagenesis of the ZMYND19 C-terminus defines a C-terminal degron motif. Raw data is provided for all ZMYND19 mutants detected in the stability profiling screen. (PSI, protein stability index; 1 = maximally unstable, 3 = maximally stable).

Supplementary Table 3 | A genome-wide CRISPR screen to identify the machinery required for ZMYND19 degradation. The output from the MAGeCK algorithm is shown.

Supplementary Table 4 | Stability profiling identifies additional CTLH substrates. ORF-level data showing the corrected read counts across the six stability bins for each ORF detected across wild-type (WT), MAEA KO and TWA1 KO cells.

Supplementary Table 5 | Identifying CTLH^{Muskelin} substrates by TMT mass spectrometry. Raw data for all proteins quantified is shown.

Supplementary Table 6 | Oligonucleotide sequences.

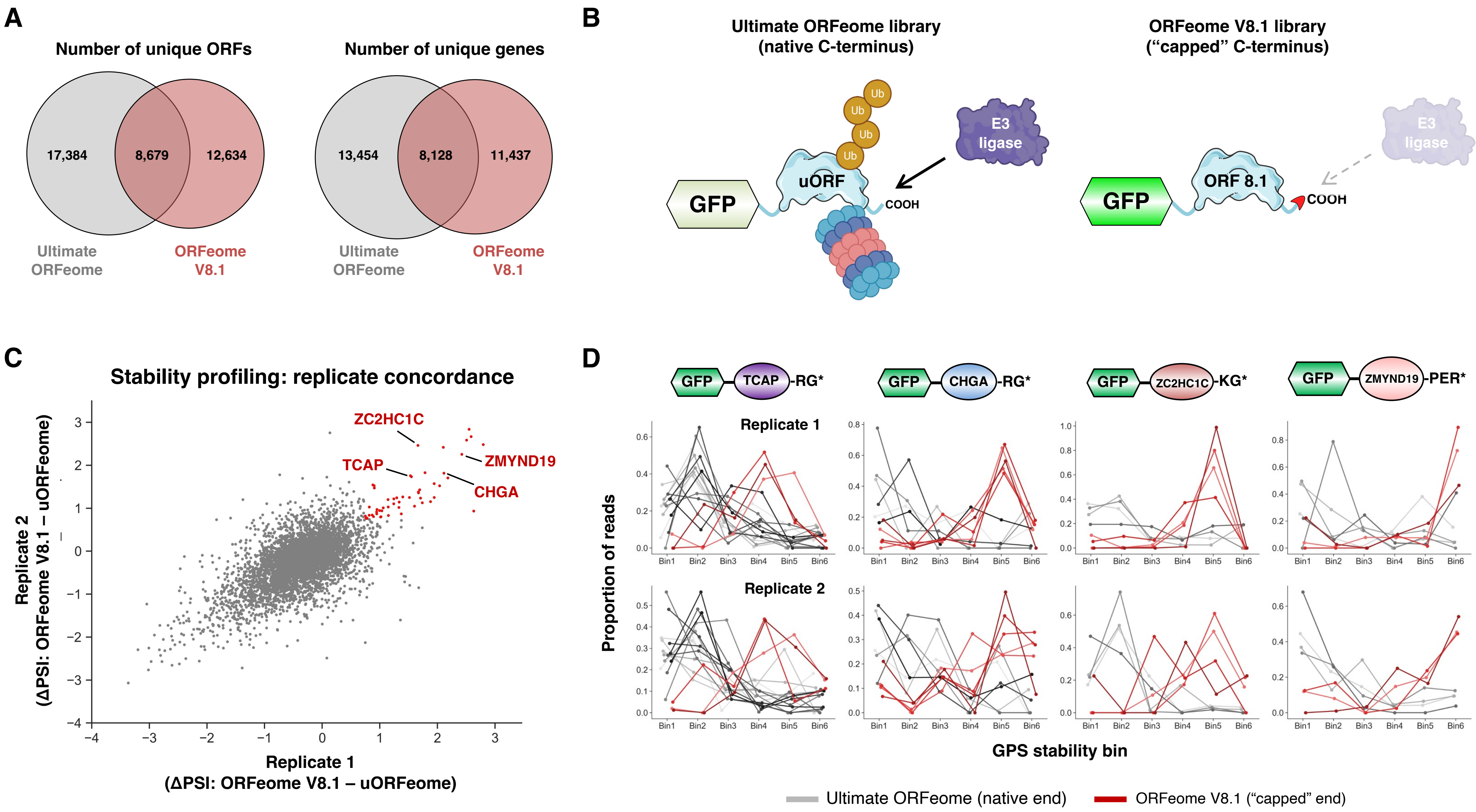
A
Number of unique ORFs
17,384
8,679
12,634
Ultimate ORFeome
ORFeome V8.1
Number of unique genes
13,454
8,128
11,437
Ultimate ORFeome
ORFeome V8.1
B
Ultimate ORFeome library (native C-terminus)
Ub
Ub
Ub
Ub
E3 ligase
GFP
uORF
COOH
ORFeome V8.1 library (“capped” C-terminus)
E3 ligase
GFP
ORF 8.1
COOH
C
Stability profiling: replicate concordance
ZC2HC1C
ZMYND19
TCAP
CHGA
Replicate 2 (ΔPSI: ORFeome V8.1 – uORFeome)
Replicate 1 (ΔPSI: ORFeome V8.1 – uORFeome)
D
GFP TCAP -RG*
GFP CHGA -RG*
GFP ZC2HC1C -KG*
GFP ZMYND19 -PER*
Replicate 1
Replicate 2
Proportion of reads
Bin1 Bin2 Bin3 Bin4 Bin5 Bin6
GPS stability bin
Ultimate ORFeome (native end)
ORFeome V8.1 (“capped” end)

Fig. S1

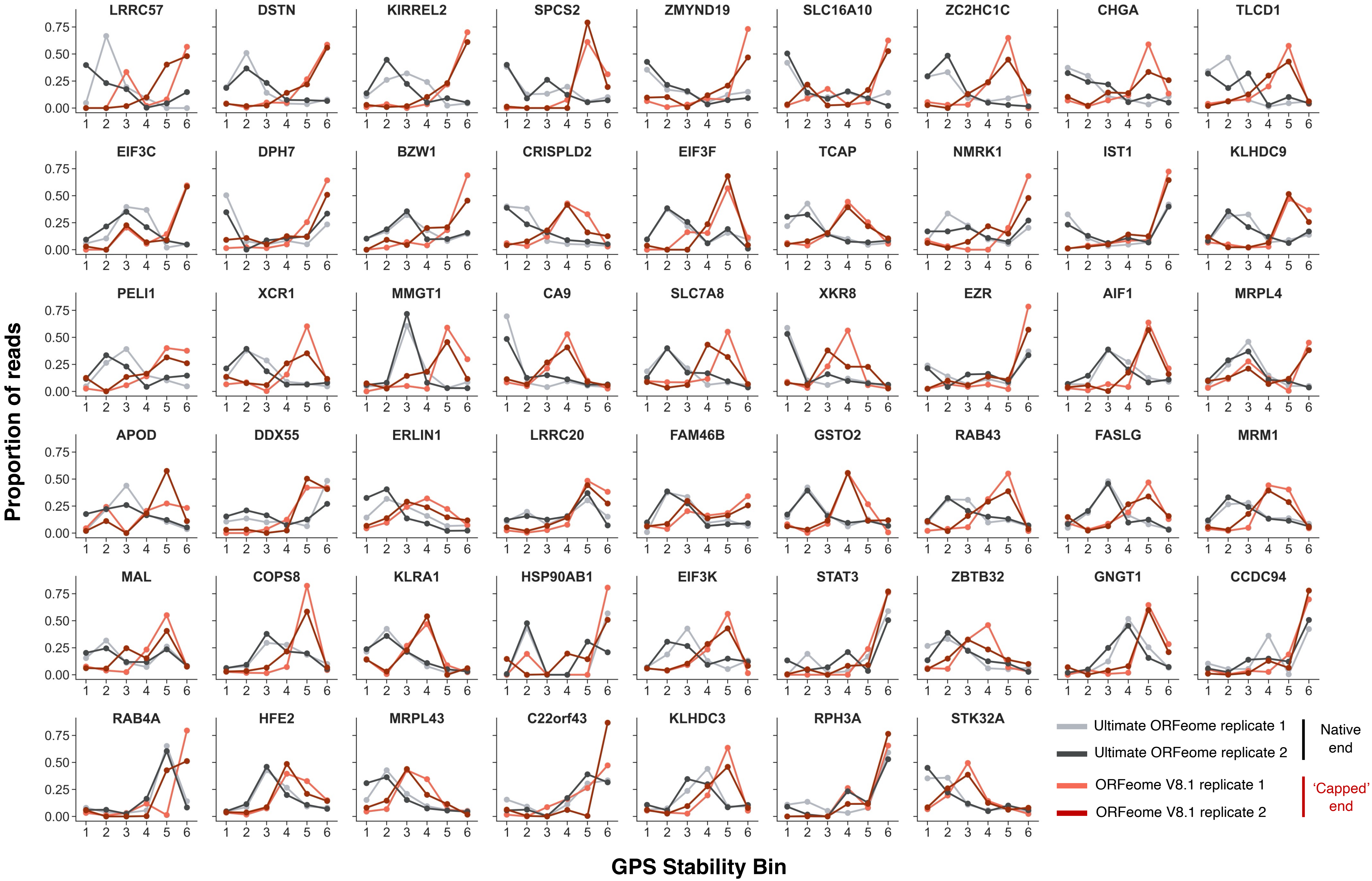

LRRC57
DSTN
KIRREL2
SPCS2
ZMYND19
SLC16A10
ZC2HC1C
CHGA
TLCD1
EIF3C
DPH7
BZW1
CRISPLD2
EIF3F
TCAP
NMRK1
IST1
KLHDC9
PELI1
XCR1
MMGT1
CA9
SLC7A8
XKR8
EZR
AIF1
MRPL4
APOD
DDX55
ERLIN1
LRRC20
FAM46B
GSTO2
RAB43
FASLG
MRM1
MAL
COPS8
KLRA1
HSP90AB1
EIF3K
STAT3
ZBTB32
GNGT1
CCDC94
RAB4A
HFE2
MRPL43
C22orf43
KLHDC3
RPH3A
STK32A
Proportion of reads
GPS Stability Bin
Ultimate ORFeome replicate 1
Ultimate ORFeome replicate 2
Native end
ORFeome V8.1 replicate 1
ORFeome V8.1 replicate 2
'Capped' end

Fig. S2

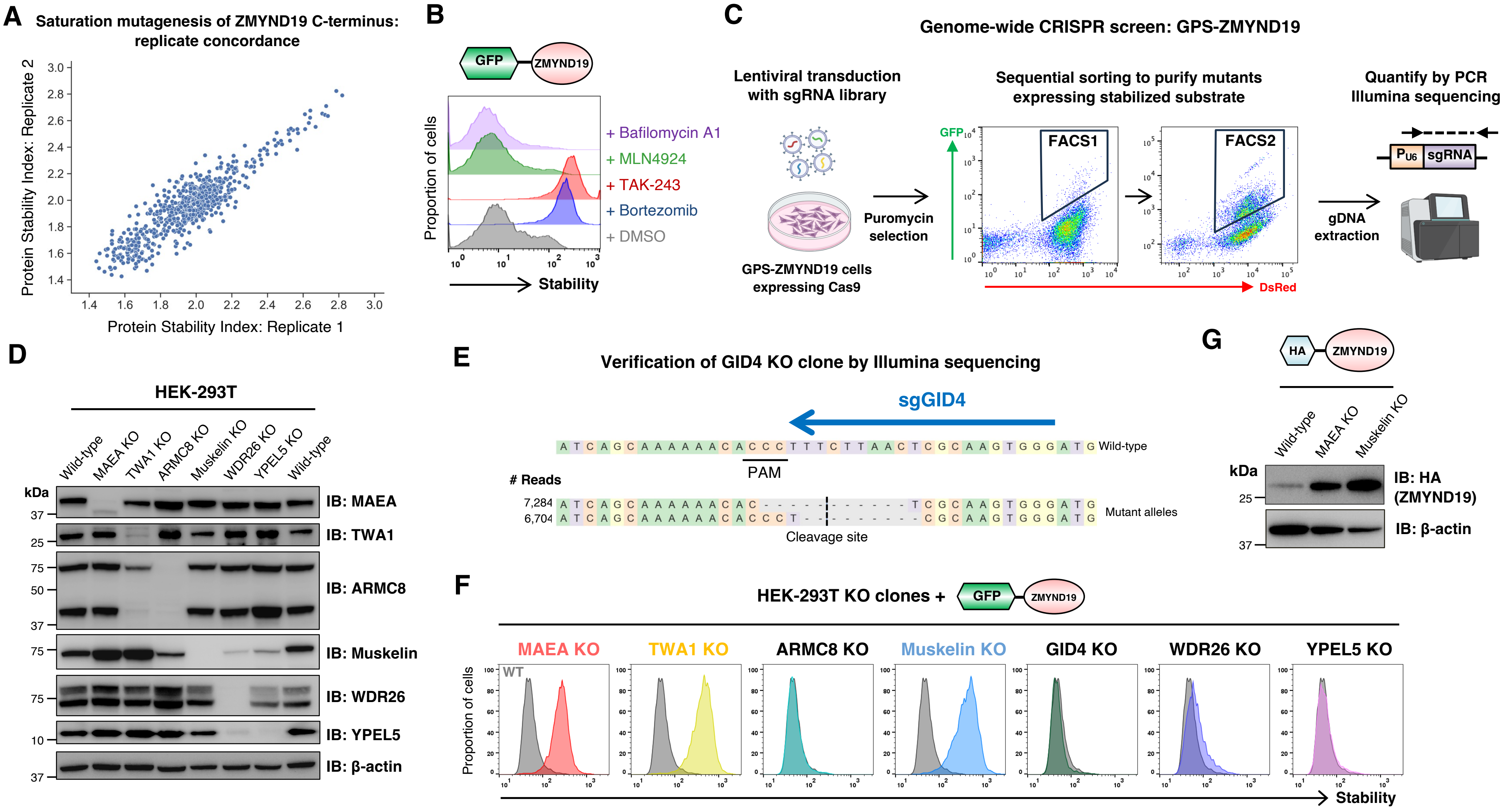
A
Saturation mutagenesis of ZMYND19 C-terminus: replicate concordance
Protein Stability Index: Replicate 2
Protein Stability Index: Replicate 1
B
GFP
ZMYND19
Proportion of cells
+ Bafilomycin A1
+ MLN4924
+ TAK-243
+ Bortezomib
+ DMSO
Stability
C
Genome-wide CRISPR screen: GPS-ZMYND19
Lentiviral transduction with sgRNA library
Sequential sorting to purify mutants expressing stabilized substrate
Quantify by PCR Illumina sequencing
Puromycin selection
GPS-ZMYND19 cells expressing Cas9
FACS1
FACS2
GFP
DsRed
gDNA extraction
sgRNA
D
HEK-293T
Wild-type
MAEA KO
TWA1 KO
ARMC8 KO
Muskelin KO
WDR26 KO
YPEL5 KO
Wild-type
kDa
IB: MAEA
IB: TWA1
IB: ARMC8
IB: Muskelin
IB: WDR26
IB: YPEL5
IB: β-actin
E
Verification of GID4 KO clone by Illumina sequencing
sgGID4
A T C A G C A A A A A A C A C C C T T T C T T A A C T C G C A A G T G G G A T G Wild-type
PAM
Reads
7,284 A T C A G C A A A A A A C A C - - - - - - - - - - - - T C G C A A G T G G G A T G
6,704 A T C A G C A A A A A A C A C C C T - - - - - - - - - - C G C A A G T G G G A T G
Mutant alleles
Cleavage site
F
HEK-293T KO clones +
GFP
ZMYND19
MAEA KO
TWA1 KO
ARMC8 KO
Muskelin KO
GID4 KO
WDR26 KO
YPEL5 KO
WT
Proportion of cells
Stability
G
HA
ZMYND19
Wild-type
MAEA KO
Muskelin KO
kDa
IB: HA (ZMYND19)
IB: β-actin

Fig. S3

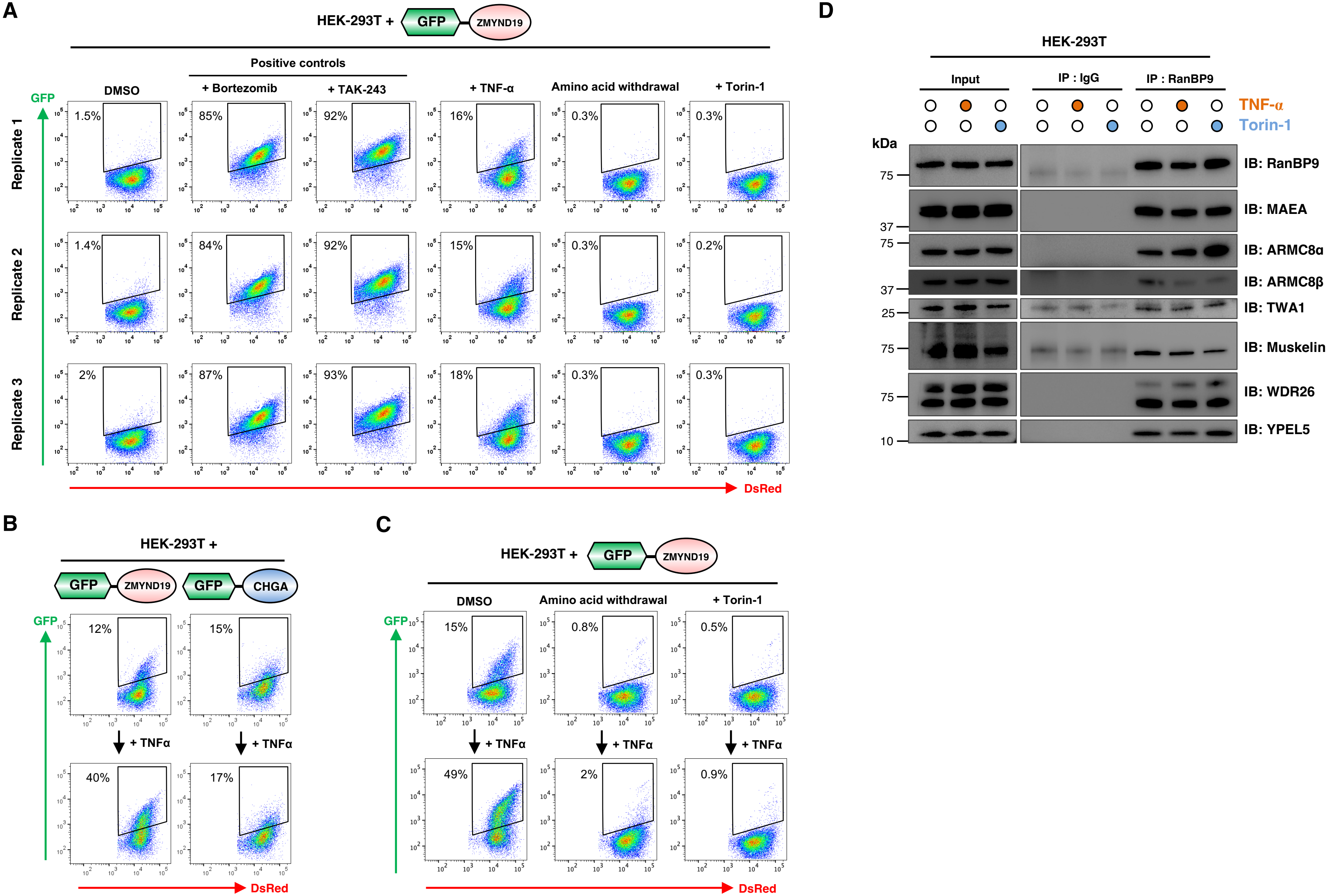
A
HEK-293T + GFP ZMYND19
Positive controls
DMSO
+ Bortezomib
+ TAK-243
+ TNF-α
Amino acid withdrawal
+ Torin-1
GFP
Replicate 1
1.5%
85%
92%
16%
0.3%
0.3%
Replicate 2
1.4%
84%
92%
15%
0.3%
0.2%
Replicate 3
2%
87%
93%
18%
0.3%
0.3%
DsRed
B
HEK-293T +
GFP ZMYND19
GFP CHGA
12%
15%
+ TNFα
40%
17%
C
HEK-293T + GFP ZMYND19
DMSO
Amino acid withdrawal
+ Torin-1
15%
0.8%
0.5%
+ TNFα
49%
2%
0.9%
D
HEK-293T
Input
IP : IgG
IP : RanBP9
TNF-α
Torin-1
kDa
IB: RanBP9
IB: MAEA
IB: ARMC8α
IB: ARMC8β
IB: TWA1
IB: Muskelin
IB: WDR26
IB: YPEL5

Fig. S4

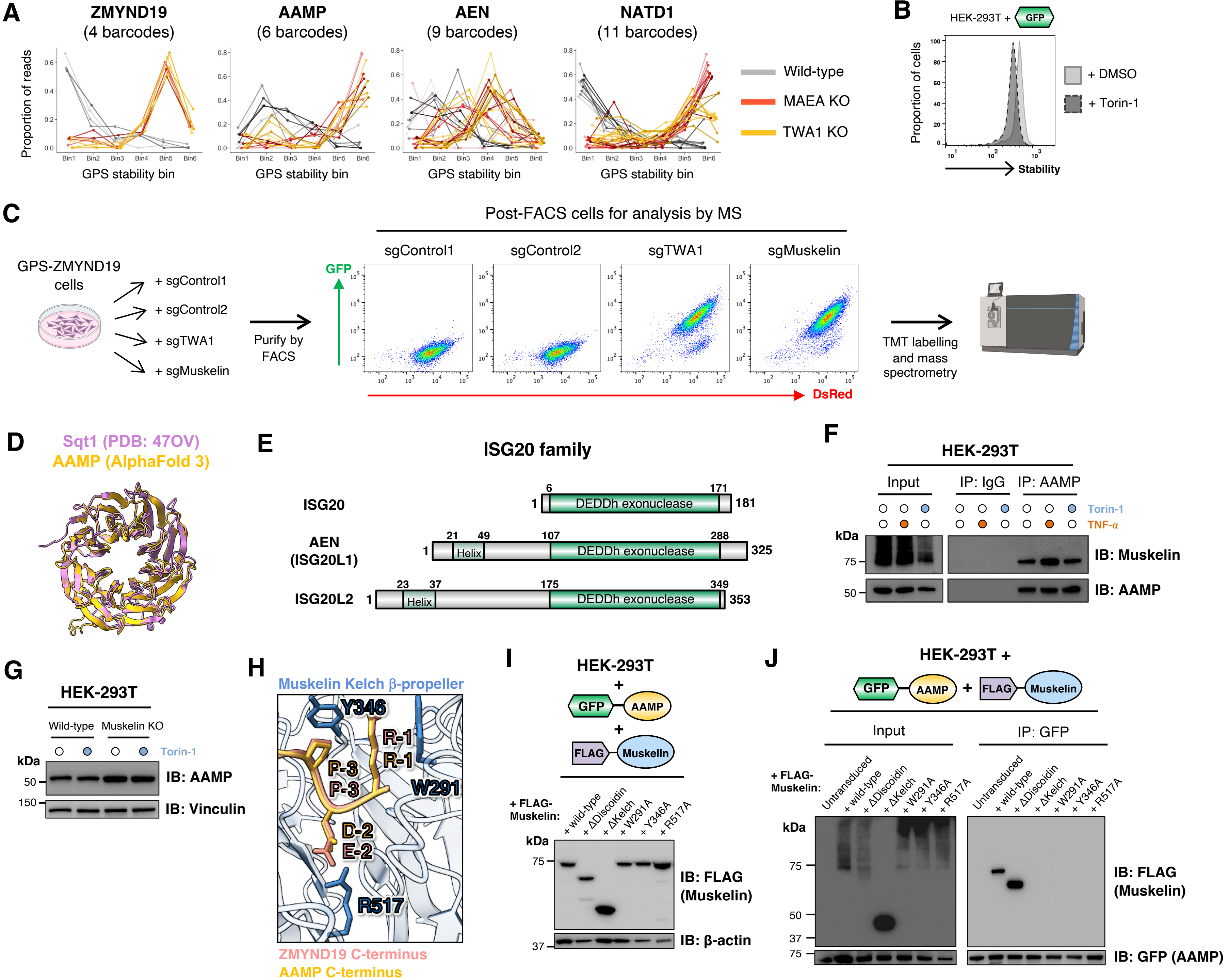
A
ZMYND19 (4 barcodes)
AAMP (6 barcodes)
AEN (9 barcodes)
NATD1 (11 barcodes)
Proportion of reads
GPS stability bin
Bin1 Bin2 Bin3 Bin4 Bin5 Bin6
Wild-type
MAEA KO
TWA1 KO
B
HEK-293T + GFP
Proportion of cells
Stability
+ DMSO
+ Torin-1
C
GPS-ZMYND19 cells
+ sgControl1
+ sgControl2
+ sgTWA1
+ sgMuskelin
Purify by FACS
Post-FACS cells for analysis by MS
sgControl1
sgControl2
sgTWA1
sgMuskelin
GFP
DsRed
TMT labelling and mass spectrometry
D
Sqt1 (PDB: 47OV)
AAMP (AlphaFold 3)
E
ISG20 family
ISG20
AEN (ISG20L1)
ISG20L2
DEDDh exonuclease
Helix
F
HEK-293T
Input
IP: IgG
IP: AAMP
Torin-1
TNF-α
kDa
IB: Muskelin
IB: AAMP
G
HEK-293T
Wild-type
Muskelin KO
Torin-1
kDa
IB: AAMP
IB: Vinculin
H
Muskelin Kelch β-propeller
Y346
R-1
R-1
P-3
P-3
W291
D-2
E-2
R517
ZMYND19 C-terminus
AAMP C-terminus
I
HEK-293T
GFP
AAMP
FLAG
Muskelin
+ FLAG-Muskelin:
+ wild-type
+ ΔDiscoidin
+ ΔKelch
+ W291A
+ Y346A
+ R517A
kDa
IB: FLAG (Muskelin)
IB: β-actin
J
HEK-293T +
GFP
AAMP
FLAG
Muskelin
Input
IP: GFP
+ FLAG-Muskelin:
Untransduced
+ wild-type
+ ΔDiscoidin
+ ΔKelch
+ W291A
+ Y346A
+ R517A
kDa
IB: FLAG (Muskelin)
IB: GFP (AAMP)

Fig. S5

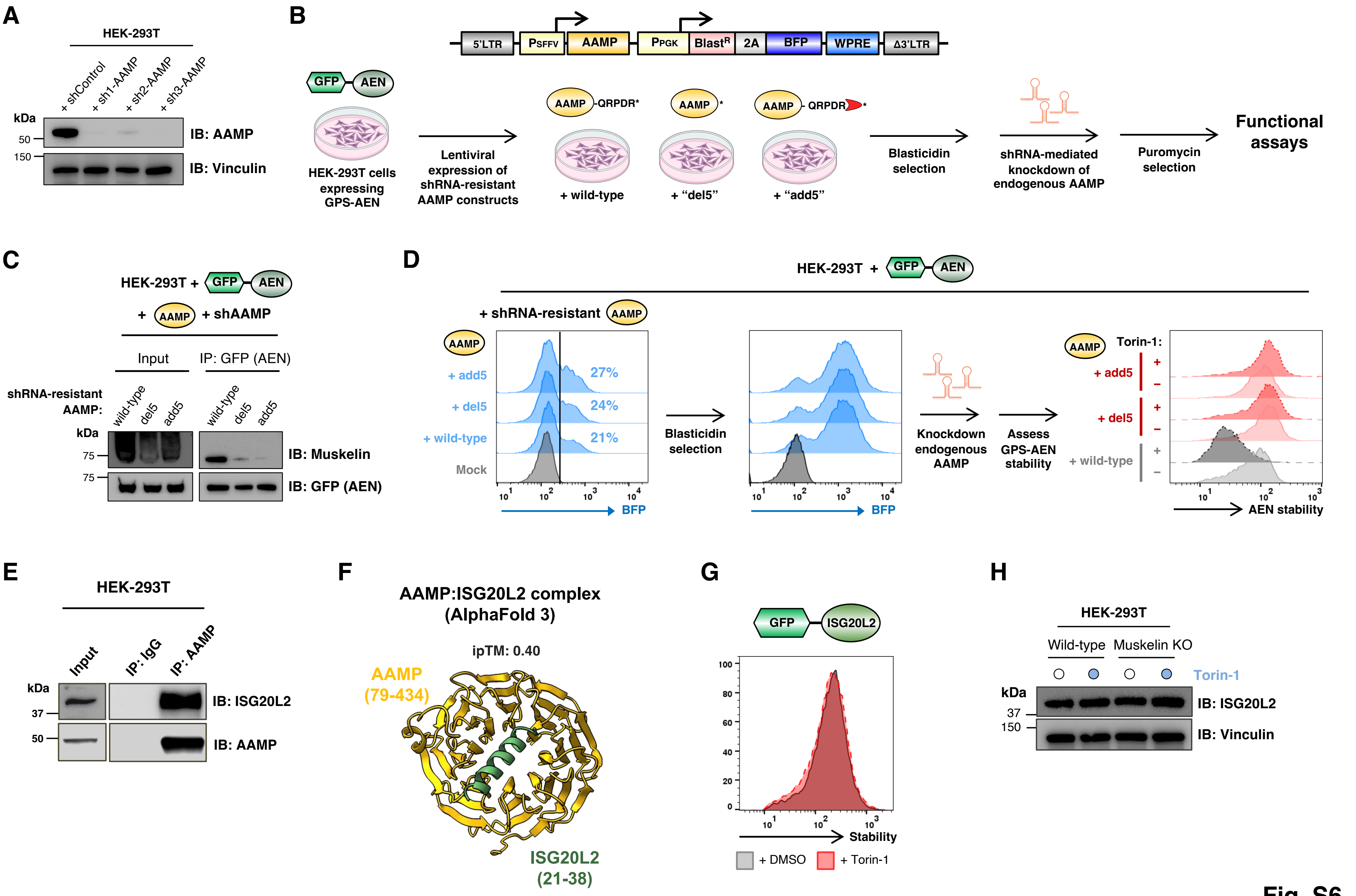

A
HEK-293T
+ shControl
+ sh1-AAMP
+ sh2-AAMP
+ sh3-AAMP
kDa
50
150
IB: AAMP
IB: Vinculin
B
5'LTR
PSFFV
AAMP
PPGK
BlastR
2A
BFP
WPRE
Δ3'LTR
GFP
AEN
HEK-293T cells expressing GPS-AEN
Lentiviral expression of shRNA-resistant AAMP constructs
AAMP
QRPDR*
+ wild-type
+ "del5"
+ "add5"
Blasticidin selection
shRNA-mediated knockdown of endogenous AAMP
Puromycin selection
Functional assays
C
HEK-293T +
+ shAAMP
Input
IP: GFP (AEN)
shRNA-resistant AAMP:
wild-type
del5
add5
kDa
75
IB: Muskelin
IB: GFP (AEN)
D
HEK-293T +
+ shRNA-resistant
+ add5
+ del5
+ wild-type
Mock
27%
24%
21%
BFP
Blasticidin selection
Knockdown endogenous AAMP
Assess GPS-AEN stability
Torin-1:
AEN stability
E
HEK-293T
Input
IP: IgG
IP: AAMP
kDa
37
50
IB: ISG20L2
IB: AAMP
F
AAMP:ISG20L2 complex (AlphaFold 3)
ipTM: 0.40
AAMP (79-434)
ISG20L2 (21-38)
G
GFP
ISG20L2
Stability
+ DMSO
+ Torin-1
H
HEK-293T
Wild-type
Muskelin KO
Torin-1
kDa
37
150
IB: ISG20L2
IB: Vinculin

Fig. S6

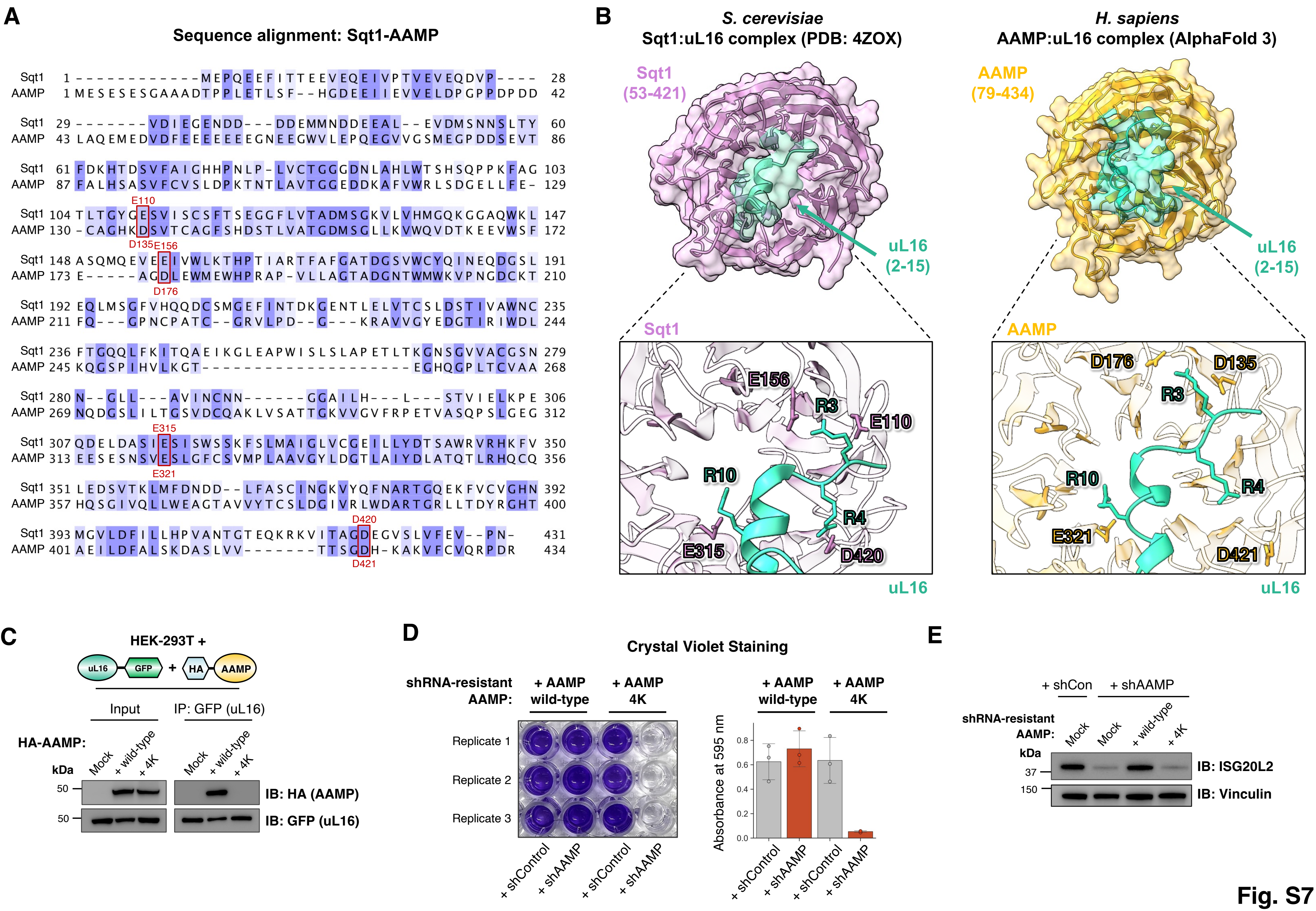
A
Sequence alignment: Sqt1-AAMP
Sqt1 1 -------------MEPQEEFITTEEVEQEIVPTVEVEQDVP---- 28
AAMP 1 MESESESGAAADTPPLETLSF--HGDEEIIEVVELDPGPPDPDD 42
Sqt1 29 -------VDIEGENDD---DDEMMNDDEEAL--EVDMSNNSLTY 60
AAMP 43 LAQEMEDVDFEEEEEEEGNEEGWVLEPQEGVVGSMEGPDDSEVT 86
Sqt1 61 FDKHTDSVFAIGHHPNLP-LVCTGGDNLAHLWTSHSQPPKFAG 103
AAMP 87 FALHSASVFCVSLDPKTNTLAVTGGEDDKAFVWRLSDGELLFE- 129
E110
Sqt1 104 TLTGYGESVISCSFTSEGGFLVTADMSGKVLVHMGQKGGAQWKL 147
AAMP 130 -CAGHKDSVTCAGFSHDSTLVATGDMSGLLKVWQVDTKEEVWSF 172
D135
E156
Sqt1 148 ASQMQEVEEIVWLKTHPTIARTFAFGATDGSVWCYQINEQDGSL 191
AAMP 173 E-----AGDLEWMEWHPRAP-VLLAGTADGNTWMWKVPNGDCKT 210
D176
Sqt1 192 EQLMSGFVHQQDCSMGEFINTDKGENTLELVTCSLDSTIVAWNC 235
AAMP 211 FQ---GPNCPATC--GRVLPD--G---KRAVVGYEDGTIRIWDL 244
Sqt1 236 FTGQQLFKITQAEIKGLEAPWISLSLAPETLTKGNSGVVACGSN 279
AAMP 245 KQGSPIHVLKGT----------------------EGHQGPLTCVAA 268
Sqt1 280 N--GLL---AVINCNN------GGAILH---L---STVIELKPE 306
AAMP 269 NQDGSLILTGSVDCQAKLVSATTGKVVGVFRPETVASQPSLGEG 312
E315
Sqt1 307 QDELDASIESISWSSKFSLMAIGLVCGEILLYDTSAWRVRHKFV 350
AAMP 313 EESESNSVESLGFCSVMPLAAVGYLDGTLAIYDLATQTLRHQCQ 356
E321
Sqt1 351 LEDSVTKLMFDNDD--LFASCINGKVYQFNARTGQEKFVCVGHN 392
AAMP 357 HQSGIVQLLWEAGTAVVYTCSLDGIVRLWDARTGRLLTDYRGHT 400
D420
Sqt1 393 MGVLDFILLHPVANTGTEQKRKVITAGDEGVSLVFEV--PN- 431
AAMP 401 AEILDFALSKDASLVV-------TTSGDH-KAKVFCVQRPDR 434
D421
B
S. cerevisiae
Sqt1:uL16 complex (PDB: 4ZOX)
Sqt1 (53-421)
uL16 (2-15)
Sqt1
E156
R3
E110
R10
R4
E315
D420
uL16
H. sapiens
AAMP:uL16 complex (AlphaFold 3)
AAMP (79-434)
uL16 (2-15)
AAMP
D176
D135
R3
R10
R4
E321
D421
uL16
C
HEK-293T +
uL16
GFP
+
HA
AAMP
Input
IP: GFP (uL16)
HA-AAMP:
kDa
Mock
+ wild-type
+ 4K
Mock
+ wild-type
+ 4K
50
50
IB: HA (AAMP)
IB: GFP (uL16)
D
Crystal Violet Staining
shRNA-resistant AAMP:
+ AAMP wild-type
+ AAMP 4K
Replicate 1
Replicate 2
Replicate 3
+ shControl
+ shAAMP
+ shControl
+ shAAMP
+ AAMP wild-type
+ AAMP 4K
Absorbance at 595 nm
0.0
0.2
0.4
0.6
0.8
+ shControl
+ shAAMP
+ shControl
+ shAAMP
E
+ shCon
+ shAAMP
shRNA-resistant AAMP:
kDa
Mock
Mock
+ wild-type
+ 4K
37
150
IB: ISG20L2
IB: Vinculin

Fig. S7

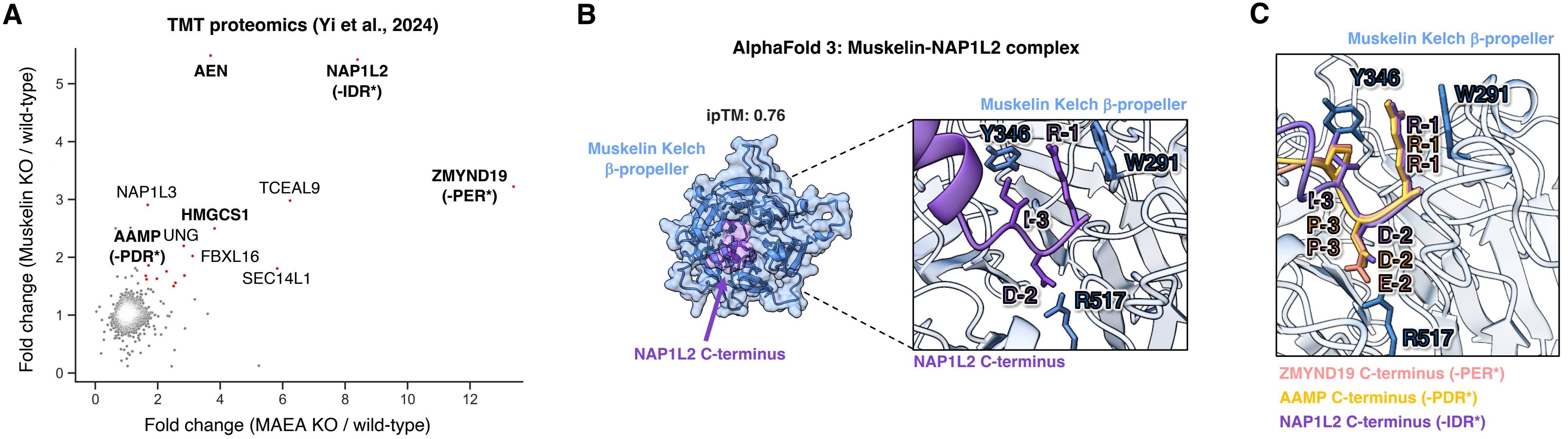

A
TMT proteomics (Yi et al., 2024)
AEN
NAP1L2
(-IDR*)
ZMYND19
(-PER*)
NAP1L3
TCEAL9
HMGCS1
AAMP
(-PDR*)
UNG
FBXL16
SEC14L1
Fold change (Muskelin KO / wild-type)
Fold change (MAEA KO / wild-type)
0
1
2
3
4
5
0
2
4
6
8
10
12
B
AlphaFold 3: Muskelin-NAP1L2 complex
ipTM: 0.76
Muskelin Kelch
β-propeller
NAP1L2 C-terminus
Muskelin Kelch β-propeller
Y346
R-1
W291
I-3
D-2
R517
NAP1L2 C-terminus
C
Muskelin Kelch β-propeller
Y346
W291
R-1
R-1
R-1
I-3
P-3
P-3
D-2
D-2
E-2
R517
ZMYND19 C-terminus (-PER*)
AAMP C-terminus (-PDR*)
NAP1L2 C-terminus (-IDR*)

Fig. S8
